# Bacteriophage Therapy for *Staphylococcus Aureus* Infections: A Review of Animal Models, Treatments, and Clinical Trials

**DOI:** 10.3389/fcimb.2022.907314

**Published:** 2022-06-17

**Authors:** Lucile Plumet, Nour Ahmad-Mansour, Catherine Dunyach-Remy, Karima Kissa, Albert Sotto, Jean-Philippe Lavigne, Denis Costechareyre, Virginie Molle

**Affiliations:** ^1^Laboratory of Pathogen Host Interactions, Université de Montpellier, CNRS, UMR 5235, Montpellier, France; ^2^Virulence Bactérienne et Infections Chroniques, INSERM U1047, Department of Microbiology and Hospital Hygiene, CHU Nîmes, Univ Montpellier, Nîmes, France; ^3^Virulence Bactérienne et Infections Chroniques, INSERM U1047, Department of Infectious and Tropical Diseases, CHU Nîmes, Univ Montpellier, Nîmes, France; ^4^Greenphage, Cap Alpha, Clapiers, France

**Keywords:** *Staphylococcus aureus*, bacteriophage therapy, case reports, clinical trials, animal models

## Abstract

*Staphylococcus aureus* (*S. aureus*) is a common and virulent human pathogen causing several serious illnesses including skin abscesses, wound infections, endocarditis, osteomyelitis, pneumonia, and toxic shock syndrome. Antibiotics were first introduced in the 1940s, leading to the belief that bacterial illnesses would be eradicated. However, microorganisms, including *S. aureus*, began to develop antibiotic resistance from the increased use and abuse of antibiotics. Antibiotic resistance is now one of the most serious threats to global public health. Bacteria like methicillin-resistant *Staphylococcus aureus* (MRSA) remain a major problem despite several efforts to find new antibiotics. New treatment approaches are required, with bacteriophage treatment, a non-antibiotic strategy to treat bacterial infections, showing particular promise. The ability of *S. aureus* to resist a wide range of antibiotics makes it an ideal candidate for phage therapy studies. Bacteriophages have a relatively restricted range of action, enabling them to target pathogenic bacteria. Their usage, usually in the form of a cocktail of bacteriophages, allows for more focused treatment while also overcoming the emergence of resistance. However, many obstacles remain, particularly in terms of their effects *in vivo*, necessitating the development of animal models to assess the bacteriophage efficiency. Here, we provide a review of the animal models, the various clinical case treatments, and clinical trials for *S. aureus* phage therapy.

## 1. Introduction

More than a century ago, bacteriophages were offered as an alternate therapy for bacterial diseases. However, the discovery of antibiotics led to phage treatment being mostly ignored, with the exception of some Eastern European nations (Chanishvili, 2012). The research and development of novel antibiotics has been largely abandoned by the majority of pharmaceutical companies (Projan, 2003; Projan and Shlaes, 2004). This has resulted in a 90 percent decrease in new systemic antibiotic approvals by the United States Food and Drug Administration (FDA) over the last 30 years (Spellberg et al., 2008; Shlaes et al., 2013). The World Health Organization (WHO) estimates that antibiotic-resistant infections account for around 700,000 deaths per year worldwide, and that this figure might rise to 10 million fatalities by 2050, accounting for more deaths than cancer (O’Neill, 2016). Therefore, the WHO has urged researchers to design innovative antibacterial approaches for treating priority antibiotic-resistant infections (Asokan et al., 2019). The use of specialized and individualized phage mixtures has shown to be an interesting alternative in the fight against multi-drug resistant bacteria (Kortright et al., 2019; AL-Ishaq et al., 2021). Phage therapy, a non-antibiotic technique for treating bacterial infections, has recently regained popularity. Phages are lytic viruses that infect bacteria from a variety of habitats, including soil, wastewater, and aquatic environments (Principi et al., 2019). Because of their specificity, the use of phages as a therapeutic option seems to be highly advantageous. Lytic phages are an acceptable option for human infection treatments as they kill their bacterial host quickly and they can be used by mixing several, minimizing the likelihood of bacteria acquiring phage resistance. Moreover, these organisms only infect their target bacteria and have no effect on the human host microbiota, in contrast to antibiotics, which can induce intestinal microbial dysbiosis (Febvre et al., 2019). Many Gram-negative and Gram-positive bacteria, especially *S. aureus*, have been effectively treated using phage treatment, although this is not yet a routine therapeutic method (Kortright et al., 2019; Luong et al., 2020). *S. aureus* is a common pathogen that causes hospital- and community-acquired infections worldwide. It is remarkably adaptable, capable of causing a wide range of illnesses varying in severity from minor skin and soft-tissue infections to lethal bloodstream infections in humans and animals (Lowy, 1998). In this review, we focus on animal models, the various clinical case treatments, and clinical trials for *S. aureus* phage therapy.

## 2. Vertebrate and Invertebrate Animal Models Developed for *S. Aureus* Phage Therapy

A number of animal models of the most prevalent and important human bacterial illnesses have been used to evaluate newly identified phages and their efficiency in combating bacterial infections *in vivo* (Melo et al., 2020). These studies have assessed the safety and efficacy of medications to translate to human therapy (Brix et al., 2020; Melo et al., 2020). Vertebrate and invertebrate animal models improve understanding of the processes of phage therapy on a living organism, including the immune response, the gastrointestinal microbiota, infected tissue, the level of security, toxicity, and potential side effects (Brix et al., 2020). Interestingly, for most of the *S. aureus*-related infections an animal model was developped, excepted to date for urinary tract infections (Luong et al., 2020). In **Table 1**, we summarized the animal model findings regarding the development of *S. aureus* phage therapy.

**Table 1:** Animal models for the development of *S. aureus* phage therapy.

Type of infection	Bacterial strain	Inoculum dose and route of infection	Phage/Cocktail	Phage dose, route of administration, and schedule	Follow-up period	Combination therapy	Results	References
**Mice, rats and rabbits**								
**Systemic infections**								
Abscess and systemic infections (mice)	*S. aureus* A170 (MRSA)	Injection of 10^6^ to 10^9^ CFU subcutaneously or intravenously	M^Sa^ phage	Injection of 10^6^ to 10^9^ PFU subcutaneously or intravenously, concurrently or 4 days later	20 days	–	Prevent abscess development, reduce mortality and bacterial clearance in blood	(Capparelli et al., 2007)
Bacteremia (mice)	*S. aureus* RCS21 (MSSA)	Injection of 2.10^8^ CFU intraperitoneally	GRCS phage	Injection of 2. 10^9^ PFU intraperitoneally after 30 min of bacterial challenge	8 days	–	Full protection from lethal bacteremia	(Sunagar et al., 2010)
Lung-derived septicemia (mice)	*S. aureus* SA27	Injection of 6,4.10^8^ CFU intranasally	S13’ phage	Injection of 10^10^ PFU intraperitoneally 6 hours postinfection	14 days	–	Significantly higher survival rates	(Takemura-Uchiyama et al., 2014)
Systemic infection (mice)	MRSA	Injection of 10^8^ CFU intravenously	*S. aureus*-specific lytic phage isolated from sewage and wastewater from Nairobi County, Kenya	Injection of 10^8^ CFU intravenously 24 or 72 hours post-infection	10 days	Clindamycin (8 mg/kg) intravenously	Treatment with phage was more effective than with clindamycin or combination treatment	(Oduor et al., 2016)
**Skin and soft tissue infections**								
Diabetic foot infection (mice)	*S. aureus* ATCC 43300 (MRSA)	Injection of 10^6^ CFU into hindpaw	MR-10 phage	Injection of 10^8^ PFU into hindpaw 30 min postinfection	12 days	Linezolid (25 mg/kg) orally	Combined bacteriophage + linezolid therapy was more effective in controlling hindpaw infection in diabetic mice versus antibiotic or phage alone	(Chhibber et al., 2013)
Diabetic foot infection (mice)	*S. aureus* Hocil17 (MSSA)	Injection of 10^7^ CFU into hindpaw	PP1493, PP1815 and PP1957 phages	Injection of 10^7^ or 10^8^ PFU into hindpaw 30 min postinfection	4 days	Linezolid (25 mg/kg) intraperitoneally	The bacteriophage assembly was more active than linezolid, which failed to resolve the infection. No antibacterial synergistic effect in combined phage + linezolid	(Albac et al., 2020)
Diabetic wound infection (mice)	*S. aureus* NSA1385 (MSSA)	Topical application of 10^8^ CFU on the wound	PN1815 and PN1957 phages	Topical application of 10^5^ PFU on the wound 48 hours postinfection	7 or 14 days	Amoxicillin-clavulanic acid (60 mg/day) orally for 5 days	Compared to treatment with systemic amoxicillin-clavulanic acid, bacteriophages had superior clinical and microbiological impact	(Huon et al., 2020)
Diabetic wound infection (mice)	*S. aureus* 63–2498 (MRSA)	Topical application of 6,7 log_10_ CFU on the wound	AB-SA01 phage cocktail (J-Sa36, Sa83, and Sa87)	Topical application of 7,9 log_10_ PFU 3, 5 and 7 days postinfection	10 days	–	Bacterial load reduction and wound closure	(Kifelew et al., 2020)
Skin and soft tissue infections (rats)	*S. aureus* ATCC 43300 (MRSA)	Injection of 10^7^ or 10^9^ CFU intramuscularly	MR-5 and MR-10 phages	Injection of 10^8^ or 10^10^ PFU intramuscularly 30 min or 12 hours postinfection	18 days	–	100% survival rate	(Chhibber et al., 2017)
Abscess infection (rabbits)	*S. aureus* 2698	Subcutaneous injection of 8.10^7^ CFU	LS2a phage	Subcutaneous injection of 2.10^9^ PFU simultaneously	4 to 6 days	–	Rabbit abscesses healed completely	(Wills et al., 2005)
**Bone and joint infections**								
Joint infection (mice)	*S. aureus* ATCC 43300 (MRSA)	Injection of 10^6^ CFU into the joint	MR-5 phage mixed with biopolymer	Injection of 10^9^ PFU into the joint followed by the infection	20 days	Linezolid mixed with biopolymer	Combined phage coating and antibiotics was effective against orthopedic implant infections	(Kaur et al., 2016)
Implant-related osteomyelitis (rats)	MRSA	Injection of 5.10^5^ CFU through the skin	Sb-1 phage	Injection of 10^7^ PFU per day through the skin for 3 consecutive days after confirmation of infection (i.e 14 days)	14 days	Teicoplanin (20 mg/kg/day) intraperitoneally for 14 days	Only bacteriophage in combination with antibiotic therapy significantly reduced bacterial load and prevented biofilm formation	(Yilmaz et al., 2013)
Periprosthetic joint infection (rats)	*S. aureus* ORI16_C02N (MSSA)	Implantation of implant pre-seeded with 1,2.10^6^ CFU into rat femur	StaPh_1, StaPh_3, StaPh_4, StaPh_11 and StaPh_16 phages	Injection of 1,3.10^8^ PFU intraperitoneally on day 21, 22, and 23 postinfection	7 days	Vancomycin (50 mg/kg) from day 21 to 27 postinfection every 12h	Treatment of infection with both vancomycin and phage significantly reduced bacterial load, while treatment with phage or vancomycin alone only caused a small reduction	(Morris et al., 2019a)
Osteomyelitis (rabbits)	MRSA	Intramedullary injection of 5.10^6^ CFU	A cocktail of seven different phages (SA-BHU1, SA-BHU2, SA-BHU8, SA BHU15 and SA-BHU21, SA-BHU37, SA-BHU47) was injected intralesionally in the infected soft tissues	Injection of 2.10^12^ PFU intraperitoneally 3 weeks postinfection with 4 doses at the interval 48h, or 6 weeks postinfection	1-4 weeks	–	Rabbits improved clinically. *S. aureus* was eradicated from acute and chronic osteomyelitis	(Abedon, 2016; Kishor et al., 2016)
**Heart and pulmonary infections**								
Lung infection (mice)	*S*. *aureus* Xen29 (MRSA)	Administration of 3.10^8^ CFU intranasally	AB-SA01 phage cocktail (J-Sa36, Sa83, and Sa87)	5.10^8^ PFU per phage intranasally at 2 and 6 hours postinfection	24 hours	–	Reduced lung bacterial burden	(Lehman et al., 2019)
Ventilator-associated pneumonia (rats)	*S. aureus* AW7 (MRSA)	Instillation of 6-8.10^9^ CFU *via* endotracheal tube	2003, 2002, 3A, and K phages	Injection of 2-3.10^9^ PFU intravenously at 2, 12, 24, 48 and 72 hours postinfection	96 hours	Teicoplanin (3 mg/kg) intravenously at 2, 12, 24, 48 and 72 hours postinfection	Significantly improved survival rates compared to absolute mortality in controls, with reduced bacterial load and better histopathological outcomes	(Prazak et al., 2019)
Endovascular infection (rats)	*S. aureus* Laus102 (MSSA)	Injection of 1,3.10^5^ CFU intravenously	vB_SauH_2002 and 66 phages	Injection of 8,2.10^10^ PFU intravenously 6 hours postinfection	24 hours	Flucloxacillin (2 g every 12 hour for 24 hours) intravenously	Phage treatment accelerated bacterial load clearance at infection sites (cardiac vegetations, blood, spleen, liver, and kidneys)	(Save et al., 2022)
Ventilator-associated pneumonia (rats)	*S. aureus* AW7 (MRSA)	Instillation of 10^10^ CFU *via* endotracheal tube	2003, 2002, 3A, and K nebulized phages	Administration of 2.10^10^ PFU directly into the lungs at 2, 12, 24, 48 and 72 hours postinfection	96 hours	Daptomycin (6 mg/kg) intravenously at 2, 12, 24, 48 and 72 hours postinfection	The combination of daptomycin and nebulized phages had saved 55% of the animals, but was not much superior to nebulized phages alone (50%)	(Valente et al., 2021)
Ventilator-associated pneumonia (rats)	*S. aureus* AW7 (MRSA)	Instillation of 10^10^CFU *via* endotracheal tube	2003, 2002, 3A, and K phages	Administration of 1,5.10^10^ PFU by inhalative treatment, intravenous treatment or a combination of both at 12, 34, 48 and 72 hours postinfection	96 hours	Linezolid (10 mg/kg) intravenously twice daily at 2, 12, 24, 48 and 72 hours postinfection	Aerophages and intravenous phages in combination saved 91% of rats from severe MRSA pneumonia in comparison to monotherapy or combination of aerophages and linezolid	(Prazak et al., 2022)
**Nematode**								
	*S. aureus* 80wphwpl	Fed for 1 day by *S. aureus* lawn	phiAGO1.3 phage	Immerged in 10^9^ PFU for 1 hours	120 hours	–	Better survival rate	(Glowacka-Rutkowska et al., 2019)
**Silkworm larva**								
	*S. aureus* SA27 and SA14	Inoculation of 10^7^-10^8^ CFU	S25-3 and S13 phages injected into the hemolymph	Injection of phage at MOI 1, 0,1, 0,01 or 0,0001 into the haemolymph 10 min, 6, 12, or 24 hours postinfection	3 days	–	No adverse effects in the silkworm larvae and life-prolonging effects	(Takemura-Uchiyama et al., 2013)

MRSA, methicillin-resistant Staphylococcus aureus; MSSA, methicillin-susceptible Staphylococcus aureus.

### 2.1 Higher Vertebrate Models

Mouse (*Mus musculus*), rat (*Rattus norvegicus*) and rabbit (*Oryctolagus cuniculus*) models have been reported for *S. aureus* phage therapy. Testing novel medicinal compounds in vertebrates is often preferred due to their genetic similarity to humans (Brix et al., 2020; Cieślik et al., 2021).

#### 2.1.1 Murine Models

Murine models are the most common for investigating *S. aureus* phage treatment in diverse pathologies as detailed below and summarized in **Table 1**. The genetic and physiological similarities between the murine and human species allow to investigate the efficacy of phage treatment developped for human medecine. Animals such as mice may only tolerate the injection of low amounts of liquid, particularly when delivered intravenously or intranasally; phages must thus be concentrated in order to accommodate the required inoculum (Penziner et al., 2021).

##### 2.1.1.1 Systemic Infections

Systemic infections are those that result in bacteremia and/or propagation to several organs. Capparelli et al. described the isolation of a phage active against local and systemic infections of *S. aureus* in mice, demonstrating the decrease in bacterial load following phage treatment (Capparelli et al., 2007). A single dose of phage treatment injected intravenously four days after the infection challenge showed a 100-fold decrease in the bacterial counts, while repeated doses resulted in a 10,000-fold decrease. Phage-treated animals were free of bacteria in their kidneys, spleens, blood, and hearts at 20 days post-therapy, while the control mouse’s organs and blood remained contaminated. They also emphasized that phage therapy administered simultaneously with bacteria inhibited the development of methicillin-resistant *S. aureus* (MRSA)-related abscesses (Capparelli et al., 2007).

Sunagar et al. (2010) developed phage therapy as an alternative treatment for fatal *S. aureus* bacteremia in diabetic and non-diabetic mouse models (Sunagar et al., 2010). In both diabetic and non-diabetic septicemic mice, a single intraperitoneal dose of the GRCS phage was significantly more beneficial than oxacillin antibiotic alone. Noteworthy, diabetic and nondiabetic mice were both given the fourth injection in a series of weekly injections of the phage GRCS. After the fourth injection, the titers of IgG and IgM antibodies against the phage rose above the background by 2500 fold and 100-fold, respectively, in both groups. Though, despite this augmentation, no allergic responses, changes in core body temperature, or other adverse effects were noted in any of the two groups (Sunagar et al., 2010). Another mouse model of bacteremia is the staphylococcal lung-derived fatal septicemia developed and tested by Takemura-Uchiyama et al., where the therapeutic benefits of phage S13′ were investigated (Takemura-Uchiyama et al., 2014). The intraperitoneal phage treatment at six hours post-infection lowered the severity of the symptoms and saved the affected mice. In addition, Oduor et al. (2016) demonstrated that a *S. aureus*-specific lytic phage found in waste and sewage water in Nairobi County could be used for MRSA bacterial infections (Oduor et al., 2016). Their mouse model provided evidence that phage treatment could be used to tackle the increasing antimicrobial resistance in sub-Saharan Africa (Lord et al., 2021).

Efficacy is often shown in studies to be dependent on the time of phage delivery. Capparelli et al. (2007) demonstrated that therapy may be postponed significantly in the case of a more chronic infection, and that the severity of the infection was dependent on the phage dosage administered. The research carried out by Caparelly and colleagues gives support to prior remarks on the limitations imposed by some experimental infection designs (Bull et al., 2002). Furthermore, in all of the publications analysed, the phages were supplied either intraperitoneally or intravenously, which are the ways *via* which drugs are frequently provided in medical practise. More research, however, is needed to evaluate other treatment options such as oral administration. As a result, the success for phage therapy in the treatment of systemic *S. aureus* infections is dependent on a number of parameters that must be extensively investigated before phage therapy can be widely used in the treatment of sepsis in humans.

##### 2.1.1.2 Skin and Soft Tissue Infections

Chhibber et al., showed that phage treatment had comparable effectiveness to linezolid antibiotic cure in halting hindpaw infection in diabetic mice with a single dose of the lytic phage, MR-10 (Chhibber et al., 2013). However, combining the treatments significantly increased the arrest of entire infection process. More recently, the same group presented a promising approach for improving phage treatment by using liposomes and transfersomes to deliver the phage to the patient (Chhibber et al., 2017; Malik et al., 2017). These vesicles disperse the substance throughout the body, avoiding fast breakdown, and increasing cellular absorption (Singh et al., 2015; Abu Lila and Ishida, 2017). In a rat model of *S. aureus* skin and soft tissue infection (SSTI), Chhibber et al.. demonstrated that transfersomes-entrapped phages injected intramuscularly generated a quicker recovery than free phages upon *S. aureus* SSTI (Chhibber et al., 2017). This novel approach demonstrates the advantages of a novel strategy to treat SSTI caused by MRSA, combining vectorization and phage delivery.

DFI are usually polymicrobial, yet, *S. aureus* is the most commonly found pathogen (Lipsky et al., 2020; Pouget et al., 2021). Recent research by Albac et al. evaluated the *in vivo* effectiveness of a cocktail of three phages (PP1493, PP1815 and PP1957) in comparison to linezolid in diabetic and non-diabetic mouse models of methicillin-susceptible *S. aureus* (MSSA) foot infection (Albac et al., 2020). In all cases, a single dose of phages into the hindpaw demonstrated considerable antistaphylococcal activity. Linezolid was as efficient as phages in non-diabetic mice but was ineffective in diabetic mice. The bacteriophages were found in high quantity in all examined organs two hours after the intravenous administration, according to the results of the pharmacokinetic data, and were still detected in the spleen 72 hours after infection, but they quickly declined in the blood, liver, and kidney, to be undetected after 48 hours (Albac et al., 2020). Moreover, during the pharmacokinetic research, no clinical symptoms of toxicity (mortality, weight loss, or decreased activity) were found in the participants overall. These results indicate that a single dose of three mixed phages at the sites of infection was about as efficient as a single dose of linezolid intravenously in lowering the bacterial load in the hindpaw of non-diabetic mice. These promising, preliminary data imply that phages may be a viable therapy option for *S. aureus* DFI that are very severe and difficult to treat. Moreover, this phage cocktail has progressed to clinical testing.

The AB-SA01 phage cocktail was applied topically to diabetic mice, where the antimicrobial activity was evaluated for its ability to heal wounds infected by MRSA clinical strains (Kifelew et al., 2020). A week after AB-SA01 treatment, a significant decrease in bacterial burdens was observed, while the non-treated group remained infected. AB-SA01 therapy may have higher or equal effectiveness to vancomycin, which is the standard first-line antibiotic for treating severe MRSA DFI. These examples of phage cocktails rather than single phage solution have the benefit of enhancing the host range of therapeutic phage compositions, and decreasing the development of phage resistant strains (Chan et al., 2013). In addition, Huon et al. (2020) used an infected diabetic wound model in mice to evaluate topical administration of phages delivered alone or in conjunction with oral amoxicillin-clavulanic acid. Clinical recovery was enhanced with phage therapy, with a decrease in local bacterial load at 7 and 14 days post-treatment (Huon et al., 2020). In comparison to antibiotic therapy, the phage medication did not have an effect on the gut microbiota. In conclusion, these latest studies demonstrate that local application of phages to cure DFI is a realistic complementary strategy when combined with oral antibiotic therapy.

Similarly to humans but in contrast to mice, rabbits are naturally susceptible to *S. aureus* infections, making them an excellent animal model for studying the development of staphylococcal diseases. A rabbit model of *S. aureus* wound infection has been designed (Wills et al., 2005) using subcutaneous injections, resulting in the formation of abscesses. Phages were given either concurrently with the bacteria or soon after at the infected region. Four to six days after infection, the phage treatment showed to be effective in preventing abscess development when the phages were administered simultaneously with *S. aureus* (Wills et al., 2005).

The studies most clearly demonstrate the safety of phage treatment when applied topically like for diabetic foot ulcer, or injected intramuscularly or in the hindpaw of mice. It is noteworthy that the majority of the studies evaluated models for phage treatment against infected diabetic foot ulcers, so proving the efficiency of such models for treating these types of infections.

##### 2.1.1.3 Heart and Pulmonary Infections

Heart and pneumonia models of phage treatment were first underrepresented in comparison to other models of infection, but they have recently gained prominence in the scientific literature as detailed below. Lehman et al. (2019) described the design of AB-SA01, a phage cocktail targeting *S. aureus*. *In vitro*, AB-SA01 was effective against 94.5% of 401 clinical *S. aureus* strains, including MRSA. Intranasal administration of AB-SA01 decreased lung *S. aureus* bacterial load to the same extent as vancomycin, in both neutropenic and immunocompetent mice models of acute pneumonia (Lehman et al., 2019). Prazak et al. (2019) investigated the effectiveness of intravenous phage therapy in ventilator-associated pneumonia (VAP) caused by MRSA (Prazak et al., 2019). In a fatal rat model of staphylococcal pneumonia, phage treatment considerably decreased mortality compared to placebo. However, there was no difference for rats treated with a combination of phages and teicoplanin. In addition, the fact that non-infected animals treated with phage showed a slight elevation in IL-1β production raises concerns regarding the use of phage treatment in the absence of a diagnosis or a strong suspicion of VAP. This effect has been reported in other investigations, which have used a variety of different experimental conditions and phages, highlighting the need for a comprehensive reconsideration of the precise influence of induced inflammation during the clinical course of infection (Van Belleghem et al., 2017). One measure that would be required would be the use of highly purified, toxin-free phage solutions that would be generated in accordance with the Good Manufacturing Practices (GMP). In a recent study by Valente et al. (2021) they determine the effects of systemic daptomycin in combination with nebulized bacteriophages in the treatment of experimental pneumonia caused by methicillin-resistant *S. aureus* (Valente et al., 2021). A rat animal model of VAP caused by MRSA was used to determine whether the simultaneous application of intravenous daptomycin and nebulised phages was superior to aerophage therapy alone in terms of improving animal survival (55 percent vs. 50%) or reducing bacterial burdens in either the lungs or an organ known to be affected by the infection. As a result, it does not seem that this combination is beneficial when used in individuals with MRSA pneumonia. However, it is still uncertain which technique of phage delivery is most effective in the setting of VAP. Therefore, Prazak et al. (2022) investigated the efficacy of aerosolized phages (aerophages) in the treatment of experimental MRSA pneumonia. Single treatment either by either aerophages or intravenous phages (IV) were able to save fifty percent of the animals who were suffering from severe MRSA pneumonia. Interestingly, aerophages and phages together saved 91% of the animals, which was a significantly larger percentage than using either treatment alone (Prazak et al., 2022). In addition, antibiotic treatment with the standard drug linezolid was successful in saving 38% of the animals, while synergy between linezolid and aerophages was not seen. However, this model exhibited significant shortcomings as it is rapidly lethal for animals and then required therapy quickly after inoculation which is not a true representation of what happens in clinical settings. The establishment of a repeatable infection also depends on a significant quantity of bacteria as well as high phage loads to produce a therapeutic effect that was satisfactory. Consequently, additional studies are required for its potential extrapolation for human treatment. Save et al. (2022) recently demonstrated the efficiency of a phage cocktail against MRSA strains tested *in vitro* and *in vivo* in a rat model of endocarditis (Save et al., 2022). Interestingly, in most studies reviewed treatment with staphylococcal phages was most effective when used in conjunction with antibiotics, confirming that this phage therapy is a promising alternative treatment.

##### 2.1.1.4 Bone and Joint Infections

There are a few animal studies on bacteriophage treatment for bone and joint infections, but most were conducted in murine models (Genevière et al., 2021; Gibb and Hadjiargyrou, 2021). As detailed in the case reports sections, implant-related staphylococcal infections resistant to most antibiotics represent a serious problem in orthopedic surgery. Yilmaz et al. (2013) investigated the effect of local application of bacteriophages against bacterial biofilms. Rats were implanted with an intravenous catheter containing a pre-generated biofilm in the tibial medullary canal and examined for implant-related osteomyelitis. The findings revealed a synergistic effect of teicoplanin and the phages in eradicating MRSA biofilms (Yilmaz et al., 2013). A similar approach was undertaken by Kaur et al. (2016), who investigated the effects of naked wire, hydroxypropylmethylcellulose (HPMC)-coated wire, or K-wire coated with phage and/or linezolid (Kaur et al., 2016). The wires were surgically inserted into the intramedullary canals of mice femora and inoculated with MRSA. The mice transplanted with K-wire coated with a combination of phage and linezolid had the greatest benefits, which included reduced inflammation of the joint and decreased bacterial adhesion to the adjacent joint tissue, as well as better recovery of limb locomotion and functional ability. Therefore, the use of dual coated implants including both linezolid and a specific MRSA lytic phage represents a novel, attractive and effective strategy in the prevention and treatment of implant-associated MRSA infections. In addition, Morris et al. (2018) previously demonstrated in *in vitro* experiments the ability of a lytic phage cocktail to lower the amount of *S. aureus* bacteria in growing biofilms on custom 3D-printed, miniaturized porous titanium implants, a material that is frequently used during for orthopedic implants (Morris et al., 2018). The same group developed a new rat model of *S. aureus* biofilm-associated prosthetic joint infection using the same titanium implants and other biomaterials used in total knee arthroplasty procedures (Morris et al., 2019b). They revealed that the combined effect of phages and vancomycin provided a considerably greater therapeutic value than separate therapy, although phage therapy alone decreased bacterial load within joint tissue and on the titanium implant of the infected knee in the first week of therapy. Furthermore, no detrimental systemic or local damage was detected after multiple doses of lytic phages containing high quantities of phages (Morris et al., 2019a). Moreover, there were no important variations in IL-6, IL-1ß, and IL-4 levels in plasma of vancomycin-treated and phage plus vancomycin-treated animals at day 28 post-surgery in comparison to treated controls.

Another study in the rabbit model found that phage treatment was successful in a MRSA osteomyelitis infection model, although others disputed the conclusions of this study (Abedon, 2016; Kishor et al., 2016). Although the viability of phage treatment was established, the rabbit model differed from the patient’s condition, in which the infection was persistent and phage medication was performed after traditional techniques had failed. This case illustrated the difficulty in developing suitable chronic models of infection. This is a recurrent issue in bacteriophage therapy as animal models are used to study acute infections, which may not be the best equivalent for phage treatment in humans, where it is used to treat chronic conditions (Kortright et al., 2019).

### 2.2 Invertebrate Models

The use of a non-mammalian model avoids the ethical issues that come with researches with in mammals. For instance, the nematode (*Caenorhabditis elegans*), common fruit fly (*Drosophila melanogaster*), wax moth (*Galleria mellonella*), silkworm (*Bombyx mori*) and zebrafish (*Danio rerio*), are among the most common invertebrate or lower vertebrate models for phage therapy (Brix et al., 2020). However only the nematode and silk worm larva models have been used for *S. aureus* studies (Takemura-Uchiyama et al., 2013; Glowacka-Rutkowska et al., 2019).

#### 2.2.1 Nematode

*C. elegans* is a small worm of 1 mm in length that may be readily infected by bacteria, fungi, and virus, resulting in deadly or non-lethal infection depending on the pathogen (Cohen and Troemel, 2015). *C. elegans* may be used for large-scale screening experiments since the pathways causing mortality in nematodes are conserved in mammals as while bypassing professional immune cells, the response against pathogens in *C. elegans* is conducted by epithelial cells that stimulate autophagy and the immune system *via* the synthesis of antimicrobial proteins, peptides (AMPs), and p38 pathway activation (Ewbank and Zugasti, 2011). As nematodes eat bacteria as their primary food source, infections may be accomplished quickly and simply. Phages can be supplied using the same mode of administration. *C. elegans* models for *S. aureus* infections and the use of phage treatment were developed recently by Glowacka-Rutkowska et al., and showed a significant decrease of mortality for larvae treated following *S. aureus* infection (Glowacka-Rutkowska et al., 2019). The staphylococcal lytic podovirus phiAGO1.3 presented a wide strain spectrum, thus demonstrating the promising potential of this phage in a clinical setting. Furthermore, they showed that phiAGO1.3 and its *S. aureus* host strains may co-exist over time, thus contributing to the emergence of phage-resistant strains but with reduced virulence (Glowacka-Rutkowska et al., 2019).

The findings of this research suggested that *C. elegans* may be used as an animal model, despite the fact that mortality was used as the only measure for analysing phage efficacy and effects.

#### 2.2.2 Silkworm Larva

As an animal model for infection, silkworm larvae offer a number of benefits, including the ability to reproduce in a short amount of time, the ability to be readily cultured in laboratories. The size of silkworm larvae is also adequate for handling during syringe injection of pathogens and therapeutics, which is a practical challenge with small-sized species such as *Drosophila melanogaster* and *Caenorhabditis elegans*. The silkworm larva has been used to test pharmacodynamics, pharmacokinetics, pathogenicity and toxicity of novel antimicrobial compounds (Kaito et al., 2002; Kaito and Sekimizu, 2007; Kurokawa et al., 2007; Fujiyuki et al., 2010). Takemura-Uchiyama et al. (2013) selected *S. aureus* phages with a wide host range from wastewater (Takemura-Uchiyama et al., 2013), isolating two staphylococcal phages, S25-3 and S13′. Administration of these phages alone had no negative impact on the silkworm larvae but had significant protective effects in silkworm larvae infected with *S. aureus*. This model was validated by comparison in an acute septic mice infection model, where the findings were comparable. Despite the distinct circulatory and immunity, the silkworm larval infection model seems relevant to test antibiotics and phage treatments upon *S. aureus* infections (Takemura-Uchiyama et al., 2013).

## 3. Case Reports of *S. Aureus* Phage Treatments

Bacteriophage treatments have been infrequently described for different infections in case studies (**Table 2**).

**Table 2:** Summary of recent published clinical reports of phage therapy in humans.

Case reports	Phage treatment and route of administration	Combination therapy	Outcomes	References
**Bone and joint infections**				
MSSA diabetic toe ulcers with osteomyelitis (n=6)	Sb-1 phage topically	–	Wounds healed without recurrence indicating successful treatment with no further antibiotic therapy (7 weeks on average)	(Fish et al., 2016)
MRSA distal phalangeal osteomyelitis (n=1)	Sb-1 phage into the soft tissue	–	Reossification of the distal phalanx (7 weeks)	(Fish et al., 2018)
MRSA and MSSA bone-related infection: pelvic bone infection (n=1), complex fracture of foot (n=1), mandibular fracture (n=1), femoral fracture under hip prosthesis (n=1), tibia osteomyelitis and fracture (n=2)	Commercially-available phage solution administered preoperatively or perioperatively *via* catheter	2 cases out of 6	In all cases, bacteriophage therapy led to complete disappearance of S*. aureus* (less than 12 months on average)	(Patey et al., 2018)
MSSA prosthetic knee-joint infection (n=4)	PP1493, PP1815, and PP1957 phage cocktail into the joint	Suppressive therapy	Beneficial with a clinically substantial improvement in function (between 3 and 18 months)	(Ferry et al., 2018; Ferry et al., 2020)
MSSA prosthetic knee-joint infection (n=1)	SaGR51ø1 phage into the joint	Cefazolin	Clinical cure, safety and lack of adverse events, with durable treatment response (6 months)	(Ramirez-Sanchez et al., 2021)
**Skin and soft tissue infections**				
MRSA infection with Netherton syndrome (congenital erythroderma) (n=1)	Pyobacteriophage cocktail and Sb-1 phage topically and orally	–	Hyperemic areas became smaller, the thickness of the yellowish film layer reduced joint mobility improved and areas of normal skin began to appear (6 months)	(Zhvania et al., 2017)
**Heart and pulmonary infections**				
*S. aureus* and *P. aeruginosa* cystic fibrosis (n=1)	Pyobacteriophage and Sb-1 phage cocktail by nebulizer	–	No adverse events and clinical response for elimination of *P. aeruginosa* and *S. aureus* (3 months)	(Kvachadze et al., 2011)
MSSA cardiomyopathy infection (n=1)	AB-SA01 phage cocktail (J-Sa36, Sa83, and Sa87) intravenously	Cefazolin, minocycline	The combined treatment resulted in negative sternal wound and intra-operative samples (approximately 3 months)	(Aslam et al., 2019)
MSSA prosthetic valve endocarditis (n=1)	AB-SA01 phage cocktail (J-Sa36, Sa83, and Sa87) intravenously	Flucloxacillin, ciprofloxacin and rifampicin	Negative blood cultures and bacteriophage infusions well-tolerated (approximately 1 month)	(Gilbey et al., 2019)
*S. aureus* cardiothoracic surgery infection (n=5)	Sa30, CH1, SCH1 or SCH111 phages, locally, orally or by inhalation	Different combined antibiotic therapy for each patient	Eradication of *S. aureus*, no severe side effects (less than 1 month)	(Rubalskii et al., 2020)
Eye, ear, nose, and throat infections				
MRSA corneal infection with chronic nasal and dermatological carriage (n=1)	SATA-8505 phage topically (eye drop, nasal spray) and intravenously	–	Negative ocular and nasal culture (3 months)	(Fadlallah et al., 2015)

MRSA, methicillin-resistant Staphylococcus aureus; MSSA, methicillin-susceptible Staphylococcus aureus.

### 3.1 Bone and Joint Infections

The rising number of patients with bone and join infections requiring extended antibiotic medication increases the risk of multidrug-resistant organism infection (Osmon et al., 2013; Schmitt, 2017). 10 to 20% of patients with periprosthetic joint infections and fracture-related infections experience treatment failure, and an even higher complication rate of 28% has been recorded for patients with foot osteomyelitis (Senneville et al., 2011; Barshes et al., 2016). However, there are limited therapeutic alternatives in treatment failure, and amputation is relatively frequent. *S. aureus* (33 to 43%), *Staphylococcus epidermidis* (18 to 40%), and *Enterococcus* species (2.5 to 15%) are the most prevalent causes of these infections (Genevière et al., 2021; Gibb and Hadjiargyrou, 2021).

A commercially available solution of Staphylococcal phage Sb-1 was used in nine patients with diabetic toe ulcers presenting *S. aureus*-bone and soft tissue infections unresponsive to antibiotic treatment, two of whom had osteomyelitis (Fish et al., 2016). These non-healing wounds with persistent osteomyelitis resolved in around 6 weeks, and a unique application of the Staphylococcal phage solution was shown to be both effective and safe in comparison to existing treatments. Osteomyelitis and cellulitis symptoms recovered promptly, and the ulcers closed without recurrence (Fish et al., 2016).

A subsequent study by the same investigators reported a MSSA diabetic ulcer and a distal phalangeal osteomyelitis in a 63-year-old female (Fish et al., 2018). The bacteriophages were applied once weekly for six weeks, with increasing radiographic reossification of the distal phalanx and reduced erythema and edema that continued to heal after the injection therapy was stopped. Therefore, such phage treatment may be beneficial in treating diabetic foot osteomyelitis (Fish et al., 2018).

Patey et al. (2018) reported multiple cases of phage-treated bone infections. These bone infections ranged from pelvic bone infection, complex foot fracture, jaw fracture, osteosynthesis and fistulized infection, femoral fracture under hip prosthesis, knee prosthesis infection, osteomyelitis of the tibia and operated tibia. Interestingly, phage treatment cured these diseases, and the researchers found that applying bacteriophages locally was safe (Patey et al., 2018).

Ferry et al. described the case of an 80-year-old patient with acute postoperative MSSA infection that was unsuccessfully treated with debridement and antibiotics. An implant retention procedure involving debridement and antibiotics was carried out, followed by injection of a cocktail of *Pseudomonas aeruginosa* and *S. aureus* phages into the joint cavity. No clinical evidence of chronic infection was seen 18 months later (Ferry et al., 2018). More recently, Ferry et al. reported a phage therapy during a debridement and implant retention procedure followed by antibiotic treatment on three patients with relapsing *S. aureus* prosthetic knee infections for whom removal of the implant was not possible (Ferry et al., 2020). After this surgery procedure and joint closure, the surgeon injected a phage cocktail directly into the joint. There was a statistically significant and clinically noteworthy increase in knee function, implying that this technique contributed to the clinical outcomes.

In 2021, Ramirez-Sanchez et al. reported the case of a 61-year-old woman treated with phages to cure a persistent MSSA prosthetic knee-joint infection. The authors noted the safety and effectiveness of their phage therapy administered intravenously and intra-articularly, as well as success using a single lytic phage (Ramirez-Sanchez et al., 2021).

Therefore, considering the bacteriophage antimicrobial efficacy and reports of its successful usage in the treatment of bone and joint infections, the possibility of using phages to develop antibacterial treatments seems promising.

### 3.2 Skin and Soft Tissue Infections

Infection of the epidermis, dermis, subcutaneous tissue, superficial fascia, or muscles may cause skin and soft tissue infections (SSTIs), which can have a broad variety of symptoms, causative agents, and severity (Ki and Rotstein, 2008). SSTIs have been further complicated due to the emergence of MRSA strains, necessitating the reevaluation of phage therapy.

Zhvania et al. (2017) reported the case of a 16-year-old boy with classic Netherton syndrome, an autosomal recessive illness including congenital ichthyosiform erythroderma, trichorrhexis invaginatus, and atopic diathesis, often associated with bacterial infections (Altman and Stroud, 1969; Zhvania et al., 2017). The patient presented atopic diathesis and recurring major staphylococcal infections as well as allergy to several medications. All treatments had been exhausted (Zhvania et al., 2017). Within 7 days of therapy with pyobacteriophage and Sb-1 phage cocktail solutions applied externally, in both liquid and cream forms, or as oral medication, this patient experienced considerable health improvement. After 6 months of therapy, his quality of life was greatly improved, and he continued the use phage at home (Zhvania et al., 2017). Therefore, phage therapy could be an effective therapeutic option for treating chronic infection for patients with recurrent infections.

### 3.3 Heart and Pulmonary Infections

Patients who have had cardiothoracic surgery are at high risk of life-threatening infections, and deaths after surgery are significantly influenced by surgical site infections. Implant-associated infections frequently become chronic because bacteria on implant surfaces tend to develop biofilms that are extremely resistant to antibiotics. *S. aureus* is one of the most common pathogens associated with infective endocarditis and its frequency has been growing in recent years (Asgeirsson et al., 2018).

Aslam et al. (2019), used phage therapy for the first time as an adjuvant to antibiotics to treat left ventricular assist device infection (Aslam et al., 2019)*. S. aureus* device infection in a 65-year-old male patient with nonischemic cardiomyopathy led to numerous hospitalizations, surgical debridements, and long-term injectable antibiotics. This patient’s continuing recurrent infections disqualified him for heart transplant. Anti-staphylococcal phage cocktail AB-SA01 was administered intravenously every 12h for 28 days, along with cefazolin every 8h and minocycline twice daily orally. The patient’s condition improved, and his sternal cultures were negative for MSSA at the end of the first week and throughout the rest of the treatment. This approach resulted in less purulence and healthier granulation tissue in the wound. Therefore, this patient could be transplanted and 7 months later was in good health with no return of infection.

Gilbey et al. (2019) recently reported the success of the first intravenous use of the staphylococcal phage cocktail ABSA01 for treating severe staphylococcal sepsis with prosthetic valve endocarditis (Gilbey et al., 2019). *S. aureus*-induced infectious endocarditis has a high mortality rate (Asgeirsson et al., 2018). A 65-year-old man with a mechanical aortic valve suffered from persistent MSSA infection on his implant. The patient recovered with AB-SA01 phage cocktail injection intravenously twice a day for 14 days in combination with antibiotics. After receiving bacteriophage infusions, no fever, tachycardia, hypotension, or rashes were seen, and no adverse sequelae were associated with the treatment.

Rubalskii et al. (2020) described a series of cases in which multi-drug-resistant or persistent infections linked with implants or transplants were effectively treated with different bacteriophages (Rubalskii et al., 2020). They developed a novel technique of individualized phage therapy in conjunction with fibrin glue composed of fibrinogen and thrombin and used a hemostat, sealant, and tissue adhesive. Phage suspension was used as a nontoxic matrix and surgically administered, allowing infected areas to receive phages for an extended period of time. Infections were completely eradicated in 7/8 patients, and there were no serious adverse side effects reported. Therefore, this innovative phage treatment method, when combined with standard antibiotic therapy, may successfully cure *S. aureus* or other bacterial infections associated with cardiothoracic surgery when conventional antibiotic therapy fails.

Lung infections are particularly difficult to treat in cystic fibrosis patients, where *S. aureus* colonizes the lungs, and despite treatment with antibiotics results in recurrent and relapsing infections (Goerke and Wolz, 2010). Phage administered by nebulizer to a 7-year-old girl with cystic fibrosis demonstrated favorable clinical outcome (Kvachadze et al., 2011). She had a long-term lung infection with *P. aeruginosa* and *S. aureus*, and had taken broad spectrum antibiotics for many years, with little impact on the bacterial invasion. The phage therapy was given nine times by nebulizer with a 4- to 6-weeks interval between each phage treatment. The proportion of *P. aeruginosa* fell dramatically after the first phage treatment, but the *S. aureus* load was not influenced. Therefore, Sb-1 was added, a well-studied staphylococcal phage with strong *in vitro* lytic efficacy against the patient’s colonizing bacterial strains. Following the application of this mix, the quantity of *S. aureus* was significantly reduced, remained low, without adverse effects. Notably, after several months of treatment, the bacterial level remained below the detection threshold, and antibiotics were reduced.

### 3.4 Eye, Ear, Nose, and Throat Infections

*S. aureus* is a primary pathogen in bacterial keratitis, a condition that may cause permanent visual impairment (Schaefer et al., 2001). Fadlallah et al. (2015) described the case of a 65-year-old woman with a corneal ulcer with interstitial keratitis in her left eye (Fadlallah et al., 2015). The patient accepted phage therapy at the Phage Therapy Center (Tbilisi, Georgia) using the *S. aureus* bacteriophage SATA-8505 after having experienced persistent nasal, dermatological, and ocular vancomycin-intermediate *S. aureus* (VISA) infection for 11 years. The patient received topical (eye drops and nasal spray) and systemic (intravenous) phage therapy treatment for 4 weeks. After 3 months, normal ocular and nasal cultures were verified, indicating eradication of the infection. Therefore, this case suggests that phage eye-drops combined to systemic phage administration may be an interesting therapeutic option for the treatment of infectious keratitis related to antibiotic-resistant *S. aureus* infections.

## 4. Clinical Trials of *S. Aureus* Phage Therapy

Beyond compassionate circumstances, the major obstacle to phage treatment is the lack of laws and policies around therapeutic application and deployment. Furthermore, the lack of clinical trials hampers the development of phage. Clinical trial methodology for phage treatment is similar to classical medication clinical trial design (Payne and Jansen, 2003). Only a few past and on-going clinical trials involving monotherapy or combination therapy approaches towards *S. aureus*-related infections have been described, as detailed in **Table 3**.

**Table 3:** Clinical trials of *S. aureus* phage therapy.

Infection	Trial	Treatment group	Placebo or comparison group	Outcomes	References
**Past**					
Chronic venous leg ulcers infected by *S. aureus*, *P. aeruginosa* and *E. coli*	Prospective, randomized, double-blind controlled study of safety and efficacy	42 individuals received WPP-201 cocktail topically	Sterile saline solution	Safety confirmed and phages did not deleteriously affect wound healing (3-6 months)	clinicaltrials.gov; #NCT00663091 (Rhoads et al., 2009)
*S. aureus* chronic rhinosinusitis	Phase 1 investigator-initiated study to evaluate the safety, tolerability and preliminary effectiveness	9 individuals received AB-SA01 phage cocktail (J-Sa36, Sa83, and Sa87) intranasally	-	Intranasal irrigation was safe and well-tolerated, with promising preliminary efficacy results	anzctr.org.au; #ACTRN12616000002482 (Ooi et al., 2019)
**On-going**					
Prosthetic joint infections of the hip or knee by *S. aureus*, *S. epidermidis, S. lugdunensis, Streptococcus* sp.*, Enterococcus faecium, Enterococcus faecalis, E. coli, P. aeruginosa*, and/or *K. pneumoniae*	Randomized open-label, parallel group, controlled study to evaluate safety and surgery sparing effect	Phage therapy in combination with antibiotics	Standard care two-stage exchange arthroplasty with antibiotics	–	clinicaltrials.gov; #NCT04787250
Second degree burn wounds prevention or infection with *S. aureus*, *P. aeruginosa* or *Klebsiella pneumonia*	Randomized, open-label, active controlled study to evaluate safety and tolerability	SPK cocktail (14 phages) topically	-	-	clinicaltrials.gov; #NCT04323475
Diabetic foot ulcers infected by *S. aureus*	Randomized, multi-center, controlled, 2-parallel-group, double-blind, superiority trial, for comparison of the efficacy	Phage solution topically	Placebo solution	-	clinicaltrials.gov; #NCT02664740

The first small phase I clinical trial was of a phage cocktail (WPP-201) in patients with venous leg ulcers (VLUs) with or without current symptoms of infection (Rhoads et al., 2009). The cocktail of bacteriophages tested was designed to selectively target particular members of the wound bacterial population, corresponding to *P. aeruginosa*, *S. aureus*, and *E. coli*, to enhance wound healing. Of the 42 patients with VLUs included, 39 completed the study successfully, while 3 dropped out. The ulcers were treated for 12 weeks with either saline or phages solution, with follow-up until 24 weeks. There were no serious side effects linked to the trial, and no statistical difference between the test and control groups for the number of side effects, the pace or frequency of healing. Therefore, the use of this phage cocktail therapy was found to be successful and safe. This cocktail now needs to be tested in phase II efficacy research with a larger sample size.

Infections with *S. aureus* are linked with persistent chronic rhinosinusitis (CRS). In a phase 1 trial, Ooi et al. (2019) studied the effectiveness and safety of an increasing dosage of the phage cocktail AB-SA01 administered intranasally to nine patients with CRS testing positive for *S. aureus* (Ooi et al., 2019). Three groups (with three patients per dose) were treated with AB-SA01 twice a day. The intranasal phage therapy was well tolerated, with no fatalities recorded in any of the groups. Eradication of infection was observed in two of nine patients indicating that the treatment was effective. This study confirmed that phage therapy might be used as a substitute to antibiotics in the treatment of people suffering from CRS. However, further research must be conducted to discover the appropriate dose and verify the effectiveness of AB-SA01 in a randomized trial.

Other phage trials are on-going, as detailed in **Table 3**. These studies include testing the effectiveness of phage treatment in preventing operation in individuals with hip/knee prosthetic joint infections; or tolerability of phage cocktail SPK as an adjuvant to conventional therapy for the management and cure of burns. Another study aims to evaluate the effectiveness of conventional therapy combined with a topical anti-staphylococcal phage cocktail to usual care in the treatment of acute and chronic ulcers infected by MRSA or MSSA.

## 5. Discussion and Concluding Remarks

This review emphasized the importance of phage therapy in the treatment of *S. aureus*-related infections and detailed the studies already performed, ranging from case reports to clinical trials, as well as the development of various animal models. Antibiotic resistance in *S. aureus* is increasing at an alarming rate, necessitating alternative therapies. Phages possess the necessary features for human therapeutic procedures, and progress has been made in testing them in clinical trials and compassionate research investigations. The versatility of phage therapy makes it an excellent choice for integration in complex and multifaceted measures to overcome staphylococcal infections. A high effectiveness level towards different strains of *S. aureus*, including MRSA, was shown in many of the studies. They were safe, as evidenced by the lack of adverse effects in most investigations. Despite the fact that numerous doses, phage delivery methods, and infection models were used in the trials, no negative side results were noted. Interestingly, these investigations showed that the antibacterial effect may be increased by improving phage administration, the use of phage cocktails, or combining them with antibiotics, as well as, using them in preventive treatments. Moreover, it is also necessary to have reglemented phage production processes, whether they are single or phage cocktails, in order to ensure their safe clinical use as the effectiveness of phage therapy is dependent in large part on maintaining phage stability and reducing immune reaction from the manufacturing process to the administration. The development of the purification procedures for the delivered phages will improve their safety, so minimising side effects and immune response. Interestingly, it is possible to prepare phage cocktails and utilise them in succession in the event of phage resistance. However, the notion of phage cocktail has been questioned, notably by the Phagoburn clinical trial on *P. aeruginosa* burn-related infections (Jault et al., 2019). Although, the use of a preassembled cocktail was required for PhagoBurn, stability issues associated with such a complex product caused differing opinions among industry experts and active regulatory agencies (ANSM, AFMPS, European Medicines Agency, and the United States Food and Drug Administration). Another clinical trial intended to illustrate the potentials of a novel kind of treatment for paediatric diarrhoea, which is a significant cause of morbidity and fatalities in Bangladesh and other poor countries (Sarker et al., 2016). The purpose of this randomised, double-blind, placebo-controlled study was to assess the efficacy of orally delivered *E. coli* phage in children between the ages of 4 and 60 months old who have been clinically diagnosed with enterohemorrhagic diarrhoea. Oral coliphages demonstrated a safe gut transit in children, but they were unable to improve diarrhoea outcomes (Sarker et al., 2016). Another randomised controlled trial after a pilot phase was conducted, where the purpose was to assess the effectiveness of intravesical bacteriophage therapy to normalise urine culture in comparison to intravesical placebo or conventional antibiotic treatment. In terms of effectiveness and safety, intravenous bacteriophage therapy was not inferior to the standard-of-care antibiotic treatment for the treatment of urinary tract infections (UTIs), though it was not superior to the placebo bladder irrigation treatment (Leitner et al., 2021).

The emergence of bacterial resistance to bacteriophages is a possibility, since bacteria already possess or have the capacity to evolve a variety of mechanisms for preventing viral infections (Seed, 2015). For instance, the *S. aureus* protein A located on the bacterial surface inhibits the adsorption of bacteriophages (Nordström and Forsgren, 1974). Antibiotic association with bacteriophages, the use of phage cocktails, or the delivery of a higher initial phage inoculum, may help to minimise the development of bacterial resistance to bacteriophages. If bacteriophages kill pathogens more quickly than they can reproduce, the used of a large inoculum is associated with less risk of the emergence of phage-resistant bacteria. Therefore, when selecting therapeutic phages, consideration should be given to the capacity of each phage to create bacterial resistance as well as estimating the dose required to prevent the formation of bacterial resistance.

Moreover, the immunogenicity of phages, which is the ability of phages to trigger particular immune responses that result in the generation of specific antibodies against phage antigens, is another crucial factor to consider. Phage immunogenicity in humans is a subject on which there are limited and conflicting opinions. Importantly, certain clinical findings suggest that the immunogenicity of phages varies greatly depending on the phage type, dosage, mode of administration, as well as the immunological condition of the host. In general, there was no clear relationship between the effectiveness of phage therapy and the presence of antiphage antibodies (Bruttin and Brüssow, 2005; Łusiak-Szelachowska et al., 2014; Żaczek et al., 2016; Łusiak-Szelachowska et al., 2017). More specifically to *S. aureus*, the dynamics of phage immunogenicity was investigated in a mouse model in which phages remained in the blood flow for 21–25 days and, despite the existence of antibodies against phages, these antibodies were unable to neutralise the phage-antibacterial activity (Capparelli et al., 2007).

Because this area of research is quickly progressing, some concerns need to be resolved, and research in animals represents one of the solutions. The animal research reviewed here suggested that phage treatment can be effective in a variety of *S. aureus* infection models, and established their safety. However, the development of animal models remains necessary. Therapy effectiveness can be established in invertebrate and vertebrate models less expensively, and more quickly than human trials and are ethically acceptable. Novel models could be considered for the future, such as the zebrafish, which is gaining popularity for studying host-pathogen interactions (Torraca and Mostowy, 2018; Rasheed et al., 2021). In addition to a mature innate immune system, zebrafish embryos are genetically similar to human, and their transparency make them ideal for investigating characteristics of infection mechanisms unreachable in standard animal models. Very recently, several zebrafish studies have been published for evaluating phage treatment upon bacterial infections, though none have studied *S. aureus* (Easwaran et al., 2017; Johansen et al., 2021; Sundaramoorthy et al., 2021). Bacteria can be injected into the embryo’s bloodstream alongside phages, and this treatment was effective with a better survival of the infected zebrafish embryos. Therefore, the use of lower vertebrates like zebrafish presents numerous benefits, including cheaper costs and shorter experiments.

Another important issue about *S. aureus* phage treatment is the lack of real clinical trials for *S. aureus* infections. More particularly, no randomized double-blind trials have been performed. The known treatments rely essentially on case reports or small clinical trial series. Moreover, despite our understanding of phages’ antimicrobial properties *in vitro*, we have limited data about their activities *in vivo*, particularly though clinical trials, and more data are required for their use in healthcare situations. Two recent reviews evaluating phage therapy against multidrug-resistant bacteria, including *S. aureus*, highlighted the importance of policies and regulations, as well as standardisation, at the national level, that would facilitate the incorporation of phages into clinical practises in the future (Cafora et al., 2019; El Haddad et al., 2019; AL-Ishaq et al., 2021; Al-Zubidi et al., 2019; Assafiri et al., 2021).

Furthermore, future studies are needed to evaluate if phages should be used alone, in cocktail, or in combination with antibiotics. Due to the possible synergistic impact of combination treatment demonstrated in some studies, it has the potential to be employed in clinical settings to effectively treat and prevent or minimise the development of bacterial resistance. We reviewed nine papers that determined the effects of combination antibiotics and phage treatment on a variety of different outcomes (**Table 1**). For the most part, the therapeutic result of combination therapy for *S. aureus* infections was synergistic, with a substantial increase in survival or a decrease in bacterial load concentration being seen in the majority of investigations. This demonstrates that the method of delivery, the dose, and even the antibiotic used may all have an impact on the effectiveness of a treatment. Together, these data support the notion that bacteriophage treatment does not intend to supplant antibiotics, but rather that it may be very effective when used in conjunction with antibiotics to treat *S. aureus* infections. However, there are only a few publications available, and further studies are needed to completely understand the treatment dynamics in combination therapy to be effectively employed in clinical practise.

In addition, the dose and mode of administration of bacteriophage treatment are the most important factors in achieving a satisfactory outcome. It is noteworthy to mention that phages were supplied by a variety of methods in the studies reviewed above, including subcutaneously, intraperitoneally, intravenously, intranasally, injection into the hindpaw or the joint, local topical application, and intramuscularly. As a result, choosing the most suitable course of administration method is dependent on the model and type of infection as well as the place of infection. Although there has been some progress, there is still a significant gap in knowledge about the application, feasibility, and safety of the various administration routes in humans.

Bacteriophage treatment is regarded as a highly individualised method, with each patient’s infections being targeted specifically. As a result, phages are a personalised therapeutic product that is tailored to the specific needs of each individual patient rather than a universal medical treatment. Therefore, the phagogram for each patient will become indispensable for a better personalised phage treatment, which must be feasible and interpretable like an antibiogram. Also, understanding of phage pharmacology is required for its use in healthcare situations. It is possible that the pharmacokinetics of the phage (uptake and delivery within the body) and the pharmacodynamics of the phage (toxic effects, adverse reactions, reduction of bacterial growth) will impact the outcome of phage therapy and that these aspects should be investigated further for phages that are destined to clinical usages (Payne and Jansen, 2003). To date, no such investigations were reported in the publications reviewed and will need to be addressed.

As a result of the growing issue of *S. aureus* antibiotic resistance around the globe, phage treatment seems to be an effective and safe therapy for fighting bacterial resistance. Some limits, on the other hand, may be identified. First and foremost, there is a lack of research that would thoroughly analyse the safety and effectiveness of phage treatment. A further limitation of the current literature is the absence of an established and regulated protocol for phage extraction and purification that leads to differences in the outcomes of the different studies reported. Finally, further research to characterize the host immune response dynamics upon treatment is required to assure that phage therapy is successful.

In conclusion, phage treatment has the potential to be important for protecting people from *S. aureus* infections in the future if a systemic strategy towards effectiveness and safety is developed in order to stimulate phage therapy development and acceptability as a treatment alternative.

## Author Contributions

LP and VM: conceptualization, writing. LP, NA-M, DC and VM: data curation. LP, NA-M, J-PL, DC and VM: writing – original draft preparation. LP, NA-M, CD-R, AS, J-PL, DC and VM: writing – review and editing. DC, J-PL and VM: supervision, resources, and funding acquisition. All authors contributed to the article and approved the submitted version.

## Funding

This study was supported by the University Hospital of Nîmes, and the Greenphage SAS.

## Conflict of Interest

The authors declare that the research was conducted in the absence of any commercial or financial relationships that could be construed as a potential conflict of interest.

## Publisher’s Note

All claims expressed in this article are solely those of the authors and do not necessarily represent those of their affiliated organizations, or those of the publisher, the editors and the reviewers. Any product that may be evaluated in this article, or claim that may be made by its manufacturer, is not guaranteed or endorsed by the publisher.
